# Perioperative management of patients on direct oral anticoagulants

**DOI:** 10.1186/s12959-017-0137-1

**Published:** 2017-05-15

**Authors:** Virginie Dubois, Anne-Sophie Dincq, Jonathan Douxfils, Brigitte Ickx, Charles-Marc Samama, Jean-Michel Dogné, Maximilien Gourdin, Bernard Chatelain, François Mullier, Sarah Lessire

**Affiliations:** 1 Université catholique de Louvain, CHU UCL Namur, Department of Anesthesiology, Yvoir, Belgium; 2 Namur Thrombosis and Hemostasis Center (NTHC), NAmur Research Institute of LIfe Sciences (NARILIS), Namur, Belgium; 3 Université de Namur, Department of Pharmacy, Faculty of Medecine, Namur, Belgium; 4 Université Libre de Bruxelles, Erasme University Hospital,Department of Anesthesiology, Brussels, Belgium; 5 Université Paris Descartes, Cochin University Hospital,Department of Anesthesiology and Intensive Care, Paris, France; 6 Université catholique de Louvain, CHU UCL Namur, Hematology Laboratory, Yvoir, Belgium

**Keywords:** Anticoagulants, Perioperative period, Invasive procedures, Spinal anesthesia, Emergency care, Blood coagulation test

## Abstract

Direct oral anticoagulants (DOACs) have been licensed worldwide for several years for various indications. Each year, 10–15% of patients on oral anticoagulants will undergo an invasive procedure and expert groups have issued several guidelines on perioperative management in such situations. The perioperative guidelines have undergone numerous updates as clinical experience of emergency management has increased and perioperative studies including measurement of residual anticoagulant levels have been published. The high inter-patient variability of DOAC plasma levels has challenged the traditional recommendation that perioperative DOAC interruption should be based only on the elimination half-life of DOACs, especially before invasive procedures carrying a high risk of bleeding. Furthermore, recent publications have highlighted the potential danger of heparin bridging use when DOACs are stopped before an invasive procedure.

As antidotes are progressively becoming available to manage severe bleeding or urgent procedures in patients on DOACs, accurate laboratory tests have become the standard to guide their administration and their actions need to be well understood by clinicians.

This review aims to provide a systematic approach to managing patients on DOACs, based on recent updates of various perioperative guidance, and highlighting the advantages and limits of recommendations based on pharmacokinetic properties and laboratory tests.

## Background

The number of patients receiving treatment with direct oral anticoagulants (DOACs) is increasing, as clinical trials have demonstrated non-inferiority or superiority in terms of prevention and treatment of thrombo-embolic events [1–11] compared with vitamin K antagonists (VKAs).

Rapid onset and offset of action, short half-life and predictable anticoagulant effects without the need for routine monitoring were the key strengths on which these anticoagulants have been marketed. However, the perioperative management and monitoring of DOACs has proved to be challenging, especially as antidotes were not available immediately following their introduction.

Several expert guidelines [12–14] were developed as soon as DOACs became available to help physicians manage these drugs. More recently, sub-group analyses of the phase III trials as well as results of recent clinical studies [15, 16] have influenced further guidelines [17–19]. Nowadays, around 10–15% of patients on DOACs will have to interrupt their anticoagulant before an invasive procedure every year [20, 21]. Furthermore, antidotes are gradually being licensed [22, 23]. This review aims to summarize current guidance on the perioperative management of DOACs to reflect published research. The literature search was performed in PubMed using the following keywords: perioperative, anticoagulant, dabigatran, rivaroxaban, edoxaban and apixaban. Only publications in English were considered.

## INDICATIONS FOR DOACs

Dabigatran (a direct anti-IIa inhibitor), rivaroxaban and apixaban (two direct anti-Xa inhibitors) are licensed in the European Union for the prevention of venous thromboembolism (VTE) after orthopedic surgery (hip and knee arthroplasties), for the prevention of thromboembolic events due to non-valvular atrial fibrillation (NVAF), and in the treatment or secondary prophylaxis of VTE [24–26]. Rivaroxaban is also licensed in the European Union for the prevention of atherothrombotic events after acute coronary syndrome with elevated cardiac biomarkers.

Edoxaban is a direct anti-Xa inhibitor that has recently been licensed in the European Union for the prevention of thromboembolic events due to NVAF and the treatment or secondary prophylaxis of venous thromboembolism only [27].

These anticoagulants are given at fixed doses and do not require repetitive coagulation monitoring in the routine follow-up of patients. Table 1 summarizes the pharmacokinetic properties of DOACs. Clinical features such as advanced age, decreased creatinine clearance (CrCl) and some drug-drug interactions are indications for using lower DOAC doses in patients at risk of having supra-therapeutic anticoagulant levels at normal doses [24–27].

**Table 1:** Pharmacokinetic properties of direct oral anticoagulants

	Dabigatran	Rivaroxaban	Apixaban	Edoxaban
Target	Factor lla	Factor Xa	Factor Xa	Factor Xa
Prodrug	Yes	No	No	No
Tmax (h)	1.0–3.0	2.0–4.0	3.0–4.0	1.0–2.0
Half-life (h)	12-17 h	5–9: healthy individuals 11–13: elderly	8–15: healthy individuals	10–14
Bioavailability	3–7% pH sensitive	For 2.5 mg and 10 mg: 80–100% (fasting or fed) For 15-20 mg: 66%: (fasting) almost 100% (fed)	± 50%	62%
Metabolism	Conjugation	CYP-dependent and independent mechanism	CYP-dependent mechanism (25%)	CYP-dependent (<5%) and independent mechanism (<10%)
Active metabolites	Yes - acylglucuronides	No	No	Yes (<15%)
Elimination of absorbed dose	80% renal	33% unchanged via the kidney	27% renal	50% renal
	20% bile (glucuronide conjugation)	66% metabolized in the liver into inactive metabolites then eliminated via the kidney or the colon in an approximate 50% ratio	73% through the liver, the residue is excreted by the hepatobiliary route	50% metabolism and biliary/intestinal excretion
CYP substrate	No	CYP3A4, CYP2J2	CYP3A4	CYP3A4 (<5%)
P-gp substrate	DE: Yes	Yes	Yes	Yes
BRCP substrate	No	Yes	Yes	No

Tmax: time to reach peak concentration; CYP3A4: cytochrome P450 isozyme 3A4;

P-gp: P-glycoprotein; BRCP: Breast cancer resistance protein

## PERIOPERATIVE MANAGEMENT OF DOACs

Recent publications strongly recommend the development of institutional guidelines and hospital policies for the perioperative management of DOACs [17, 18]. A checklist including all aspects of the particular procedure and the patient characteristics that may increase the risks of bleeding or thrombosis should be available to guide the perioperative use of DOACs.

Table 2 shows items to consider in a perioperative checklist.

**Table 2:** Items of the perioperative checklist

THE PERIOPERATIVE CHECKLIST	
➢ The thrombo-embolic risk of the patient	
➢ The bleeding risk of the patient	
➢ Timing of stopping DOAC before an invasive procedure: • The bleeding risk of the invasive procedure • The elimination half-life of the DOAC used depending on the patient’s ° renal function, liver function, and co-medication	
➢ Specific considerations for some invasive procedures: ° Neuraxial anesthesia ° Atrial fibrillation ablation	
➢ When should bridging therapy with heparin be suggested?	
➢ Resuming a DOAC after an invasive procedure or surgery	

### The thrombo-embolic risk of the patient

#### CHADS2 or CHA2DS2-VASc score

The CHADS2 score and the CHA2DS2-VASc score are used to predict AF-related thromboembolic risk in the absence of anticoagulation and to determine anticoagulant therapy [28, 29]. The CHA2DS2-VASc score has shown better discrimination of patients at truly low risk of thrombo-embolism (TE) [30]. Patients with chronic AF have twice the risk of postoperative stroke than patients without AF [31]. Currently, these scores are proposed to identify patients with AF at high TE risk in a perioperative setting, when they have more than 4 individual risk factors for stroke [19, 32, 33]. However, only the individual risk factors from the CHADS2 stroke risk index were assessed in stratifying the risk of postoperative stroke and not the global value of both scores. Therefore the utility of the CHADS2 and CHA2DS2-VASc scores to predict perioperative stroke needs to be prospectively validated [34].

Apart from these scores, patients with a recent history of stroke or transient ischemic attack (within 3 months) are considered as high TE risk. The 9th edition of the American College of Chest Physicians (ACCP) guidelines on perioperative management of antithrombotic therapy go further and suggest considering at high TE risk:

- Patients with AF and a prior stroke or transient ischemic attack (occurring >3 months before the planned surgery).
- Patients with a CHADS2 score < 5 having prior thromboembolism during temporary interruption of oral anticoagulants [32, 33].

#### Timing of last thrombo-embolic event

For patients with VTE, thrombosis, thrombus propagation and embolization can occur up to 12 months after diagnosis and initiation of the treatment. The ACCP guidelines consider that during interruption of anticoagulant treatment, the risk of recurrence of VTE is a high risk if the last acute VTE occurred less than 3 months ago, an intermediate risk if VTE occurred between 3 and 12 months ago, and a low risk if the last VTE occurred at least 12 months ago. The authors recommend taking individual patient factors into consideration, such as patients with remote (> 12 months ago), but severe VTE associated with pulmonary hypertension. These patients may be perceived as high risk though they would be classified as low risk [32].

Regarding patients with a VTE event <3 months, procedures requiring DOAC discontinuation during this time interval must be discussed with a multidisciplinary team to assess the urgency of the procedure. The procedure should be postponed if possible, and if not, a bridging therapy with heparin should be discussed due to the high case fatality of recurrent VTE during the initial 3 months (11.3% (CI, 8.0% to 15.2%)) [35]. Caution is advised in patients with initial symptomatic pulmonary embolism (PE) as they have a higher risk of recurrent VTE than those with initial deep vein thrombosis (DVT) without PE, and as they are at higher risk of recurrent symptomatic PE [36], with a case-fatality of 26.4% (95% CI, 16.7%–38.1%) [37].

### Other risk factors

Patients with active cancer receiving chronic anticoagulation are prone to thrombosis and bleeding complications. Tafur et al. showed that patients with active cancer in whom anticoagulation (warfarin) was temporarily interrupted for an invasive procedure had significantly higher 3-month rates of VTE, major bleeds and death. These outcomes were observed only for those cancer patients receiving anticoagulant therapy for prior VTE events, not for patients on long-term anticoagulation for AF or mechanical heart valves (MHV) where the cancer status did not affect either thromboembolic or major bleeding outcomes [38].

Kaatz et al. showed that the 30-day postoperative incidence of stroke supports the premise that the perioperative milieu is prothrombotic [39]. Prolonged bed rest in healthy volunteers does not induce a hypercoagulability state [40], however this is not generalizable to the perioperative context and caution should be advised if prolonged immobilization is required post-surgery.

The ACCP guidelines consider certain types of surgery to be associated with an increased risk for stroke or other thromboembolism (eg, cardiac valve replacement, carotid endarterectomy, and major vascular surgery) [32].

### The bleeding risk of the patient

The use of specific scores can help to assess the risk of major bleeding in patients with oral anticoagulant treatment. As these scores have never been validated in the perioperative setting to guide anticoagulant management, there is a real need to develop a bleeding risk score dedicated to surgical patients.

Different scores have been evaluated such as the **HAS-BLED score** [41], the **ORBIT bleeding risk score** [42] and the novel **biomarker-based ABC**– **bleeding risk score**. The last score performed better in predicting major bleeding in patients with atrial fibrillation than the HAS-BLED and ORBIT scores, but is probably more difficult to use in practice [43].

A high bleeding risk score should generally not suggest that oral anticoagulants are stopped, but modifiable bleeding risk factors should be identified and treated [44].

### Time of stopping DOACs before an invasive procedure

Two main characteristics influence the timing for the last DOAC administration before an invasive procedure: the bleeding risk of the procedure and the elimination half-life of the DOAC.

#### The bleeding risk of invasive procedures

Invasive procedures should be classified as low or high bleeding risk. Various classifications of procedural bleeding risks have been published [19, 33, 45, 46].

Each institution should have a detailed list of the bleeding risks of all the invasive procedures that are performed there such as that presented in Table 3.

**Table 3:** Examples of bleeding risk stratification for invasive procedures

Minimal risk of bleeding or feasible with on-therapy levels of direct oral anticoagulants^a^	Low to moderate risk of bleeding	High risk of bleeding
Tooth extraction: 1 to 3 teeth Periodontology Simple endoscopy without biopsy Superficial surgery (e.g. abscess incision or minor dermatologic procedures (small superficial excision) Cataract procedure Double J stent insertion	Endoscopy with simple biopsy Prostate or bladder biopsy Coronary angiography Simple abdominal hernia repair Anal surgery Gynecologic surgery: simple total laparoscopic hysterectomy Orthopedic surgery: hand surgery, arthroscopy Pace-maker or cardioverter-defibrillator implantation^b^	Neuraxial anesthesia Intracranial surgery Thoracic surgery Cardiac surgery Complex abdominal or gynecological cancer surgery Major orthopedic surgery Ear/Nose/Throat complex cancer surgery or specific surgery requiring good hemostasis (e.g. cochlear implant or thyroid surgery) Liver and kidney biopsy Transurethral prostate or bladder resection Extracorporeal shockwave lithotripsy Infected pace maker lead extraction (increased risk of cardiac tamponade) Robotic surgery

^a^We suggest realizing these procedures at trough levels of direct oral anticoagulants (e.g. avoiding the intake the morning of the procedure)

^b^Awaiting results of BRUISECONTROL-2 trial (NCT01675076) to decide whether device procedures can be safely realized on direct oral anticoagulants

There is sufficient evidence that some procedures may be performed on patients receiving anticoagulant therapy (i.e. procedures with minimal risk), as for example superficial skin surgeries, parietal surgery, cataract surgery, and minor dental procedures [47]. It is suggested that the morning dose of anticoagulant should be omitted on the day of the procedure to avoid peak concentrations during the procedures [19].

Pacemaker or cardioverter-defibrillator devices can be implanted safely without stopping VKAs, but more evidence is needed about DOACs [48, 49]. The BRUISE CONTROL-2 trial, which is currently recruiting, aims to show that performing device surgery without interruption of the DOAC will result in a reduced rate of clinically significant hematoma [48].

#### The elimination half-life of the DOAC

The elimination half-life of DOACs can be increased by decreased renal function (dabigatran > > edoxaban > rivaroxaban and apixaban), severe liver insufficiency (rivaroxaban and apixaban > edoxaban > dabigatran) and co-medications.

- ***Renal function***

The creatinine clearance (CrCl) must be calculated by the Cockcroft - Gault equation, as the Modified Diet in Renal Disease (MDRD) equation overestimates renal function at lower levels. Renal function should be systematically checked when underlying conditions might affect it [17].

b. ***Liver function***

None of the perioperative proposals recommend altering DOAC pre-procedural administration in cases of liver insufficiency. However, moderate to severe, chronic liver disease can increase rivaroxaban plasma concentrations. Therefore, liver function should be regularly rechecked in chronic or acute liver impairment.

c. ***Other risk factors***

Older age, extreme low body weight (<50 kg) and co-medication with important drug-drug interaction should also be researched as they can increase the half-life of DOACs or anticoagulant concentrations.

Drugs that strongly inhibit CYP3A4 and P-glycoprotein (P-gp) increase bleeding risks due to increased anticoagulation concentrations [50–52]. Anti-platelet agents, anti-inflammatory drugs, selective or non-selective serotonin reuptake inhibitors and all anticoagulants are medications that, while not directly affecting DOAC metabolism or transport, increase the bleeding risk when co-administrated with DOACs. Despite drug-drug interactions that frequently occur in patients treated with NVAF [53], only a few perioperative proposals recommend an extra delay before surgery in such cases [18].

Table 4 summarizes recent guidance from various expert groups.

**Table 4:** Summary of recent propositions for perioperative management of DOACs

DOAC			Dabigatran		Rivaroxaban - Apixaban		Edoxaban	
Bleeding risk of invasive procedure			LOW Bleeding risk	HIGH Bleeding risk	LOW Bleeding risk	HIGH Bleeding risk	LOW Bleeding risk	HIGH Bleeding risk
GIHP (Groupe d’Intérêt en Hémostase Péri-opératoire)	Preoperative interruption *No bridging (except patients with high risk of TE)*	CrCl ≥50 ml/min	Last dose ≥ 24 h before surgery	Last dose 4 days before surgery	Last dose ≥ 24 h before surgery	Last dose 3 days before surgery	Last dose ≥ 24 h before surgery	Last dose 3 days before surgery
		CrCl >30 ml/min		Last dose 5 days before surgery				
		For very high risk procedure (neuraxial anaesthesia)	Last dose 5 days before surgery					
	Resumption after invasive procedure or surgery		LOW Bleeding Risk: Resume minimum 6 h after invasive procedure or surgery HIGH Bleeding Risk: Prophylactic dose of LMWH, UFH or fondaparinux minimum 6 h after invasive procedure or surgery if venous thromboprophylaxis is indicated Therapeutic dose of DOACs when hemostasis is controlled (24-72 h) For neuraxial anesthesia with indwelling catheter: Resumption with LMWH or UFH until indwelling catheter is out					
Heidbuchel et al.	Preoperative interruption *No bridging*	CrCl ≥80 ml/min	≥ 24 h	≥ 48 h	≥ 24 h	≥ 48 h	≥ 24 h	≥ 48 h
		CrCl 50–80 ml/min	≥ 36 h	≥ 72 h	≥ 24 h	≥ 48 h	≥ 24 h	≥ 48 h
		CrCl 30–50 ml/min	≥ 48 h	≥ 96 h	≥ 24 h	≥ 48 h	≥ 24 h	≥ 48 h
		CrCl 15–30 ml/min	Not indicated	Not indicated	≥ 36 h	≥ 48 h	≥ 36 h	≥ 48 h
		CrCl <15 ml/min	No official indication for use					
	Resumption after invasive procedure or surgery		LOW Bleeding Risk: • DOACs 6-8 h HIGH Bleeding Risk: • Low TE risk ➔ resume DOACs 48-72 h after procedure • High TE risk ➔ prophylactic or intermediate dose of LMWH 6-8 h after procedure, resume DOACs when hemostasis is controlled (48-72 h)					
Spyropoulos et al.	Preoperative interruption *No bridging*	CrCl >50 mL/min	Last dose 2 days before surgery	Last dose 3 days before surgery	Last dose 2 days before surgery	Last dose 3 days before surgery	Last dose 2 days before surgery	Last dose 3 days before surgery
		CrCl 30–50 mL/min	Last dose 3 days before surgery	Last dose 4–5 days before surgery	Last dose 2 days before surgery	Last dose 3 days before surgery		
		CrCl 15–29 mL/min			Depends on patient and procedural factors	Depends on patient and procedural factors		
	Resumption after invasive procedure or surgery		LOW Bleeding Risk: • DOACs 24 h HIGH Bleeding Risk: • Low TE risk ➔ resume DOACs 48-72 h after procedure • High TE risk ➔ consider a reduced dose of dabigatran (75 mg twice daily), rivaroxaban (10 mg once daily) or apixaban (2,5 mg twice daily) on the evening after surgery and on the following day (first postoperative day) after surgery.					

CrCl: creatinine clearance, LMWH: low molecular weight heparin, UFH: unfractionated heparin, TE: thrombo-embolic

### Specific consideration for some invasive procedures

#### Neuraxial anesthesia

Two multicenter studies have published findings on residual perioperative DOAC concentrations. Both concluded that 48 h without treatment might not guarantee the absence of residual anticoagulant effect at the time of the procedure in around 15% of patients and suggested that the period of stopping a DOAC before very high bleeding risk procedures requiring complete hemostatic function such as neuraxial anesthesia or intracranial surgery should be prolonged [16, 54].

Such findings are important as some expert groups have classified these procedures as having a similar risk of bleeding as other conventional high-risk procedures and recommend a minimum 48 h of stopping DOAC treatment in patients with normal CrCl (>80 ml/min) for dabigatran and with moderate to normal CrCl (CrCl >30 ml/min) in patients on rivaroxaban, apixaban or edoxaban [45].

Yet, procedures such as neuraxial anesthesia must be considered as major bleeding risk interventions that require complete hemostatic function. The overall incidences of neuraxial hematoma in patients receiving an epidural or spinal anesthesia are estimated to be 1/220,000 and 1/320,000 patients, respectively [55]. In the presence of risk factors such as multiple attempts, spinal abnormalities, inherited or acquired coagulopathies, and heparin administration, the bleeding risk can be increased by up to two orders of magnitude (e.g. 1/3600 in the study published by Moen et al. including female patients undergoing knee arthroplasty) [55–58].

Rosencher et al. [59] and Gogarten et al. [60] advise an interruption of two half-lives before neuraxial anesthesia, but firstly this recommendation is based only on the prophylactic dosage and secondly it does not take into account the huge inter and intra-individual variability of DOAC plasma concentrations [61].

Recent guidelines published by the American Society of Regional Anesthesia (ASRA) [62] recommend an interval of 5 half-lives to allow complete elimination between stopping oral anticoagulants and carrying out medium or high-risk pain procedures [62]. Due to the variability in DOAC metabolism and elimination, this interval corresponds to 4–5 days for dabigatran, and 3 days for rivaroxaban and apixaban [57, 63]. Similarly in a very cautious approach, the GIHP proposed 5 days of DOAC interruption before neuraxial anesthesia [19].

Douketis et al. commented recently on the latest ASRA guidance. With the high inter-individual variability of DOAC plasma concentration [15, 61], they warned that estimating the elimination half-life of DOACs using the glomerular filtration rate is not sufficient to determine the required stoppage period before neuraxial anesthesia. They suggested that the ideal timing of stopping DOAC treatment should be based on residual plasma concentration measured in the perioperative setting. Benzon et al. replied that further research projects implementing DOAC perioperative measurements need to be conducted to provide sufficient high quality evidence to guide perioperative DOAC interruption. For perioperative research purposes, the use of adapted specific laboratory tests to estimate low plasma concentration of DOACs need to be used and will be discussed in a later chapter.

#### Atrial fibrillation ablation

Catheter ablation (CA) for atrial fibrillation performed by venous femoral puncture is a common procedure performed worldwide. However, this procedure can cause potentially life threatening bleeding complications such as cardiac tamponade, and also carries a specific TE risk. For periprocedural anticoagulation, undergoing CA with uninterrupted warfarin became a standard as it was associated with fewer periprocedural strokes and minor bleeding complications than low molecular weight heparin (LMWH) [64]. For uninterrupted DOACs (rivaroxaban, dabigatran and apixaban), research evidence tends to the same conclusions as with uninterrupted warfarin [65–69]. However, there is an important variability in the definition of uninterrupted DOACs in the literature. For example, uninterrupted dabigatran can be defined as a last dose on the evening before CA [66] or in the morning of the procedure [70]. Studies should use a standard protocol for uninterrupted DOACs to allow comparison between results.

Unfractionated heparin (UFH) is given during the procedure for left-sided ablation as a prophylactic measure to prevent clot formation. Activated clotting time (ACT) is used to measure UFH anticoagulation during the procedure. However, several ex-vivo and in-vitro studies have shown that ACT can be influenced by concomitant DOAC in the plasma. Indeed, some studies report that it takes more time [70] and higher doses of UFH to reach the target ACT (300–400 s) than in patients undergoing CA with uninterrupted warfarin or acenocoumarol [70–72]. Other studies report that DOACs influence ACT baseline, according to the variable sensitivity of ACT to each DOAC and to the specific instrument used to measure ACT [73]. Further studies are needed to validate the safety and the relevance of the target ACT (300–400 s) with UFH during the procedure with uninterrupted DOACs.

### When should bridging therapy with heparin be suggested?

The administration of LMWH after stopping oral anticoagulants was initially recommended to avoid a perioperative gap with insufficient anticoagulation.

The recommendations of the European Society of Anaesthesiology (ESA) published in 2013 [74] suggested that bridging therapy with heparins could be used when DOAC had a long preoperative interruption (5 days before surgery) in patients at high TE risk (for dabigatran, it considered patients with normal to mildly impaired renal function only) (grade 2C). Their categorization of patients at high risk of TE differed from the one proposed in the ACCP guidelines, as the ESA included patients with AF and CHADS2 score > 2.

Since the latter recommendations, several studies have failed to support the benefit of heparin bridging in reducing perioperative TE at DOAC arrest.

Schulman et al. published the first prospective trial of dabigatran perioperative management, without heparin bridging, except in postoperative situations where patients were fasting or when an indwelling neuraxial catheter was in place. They included 541 patients in two years and recorded only one transient ischemic attack (0.2%; 95% CI, 0–0.5). They concluded that bridging was not necessary for a standardized arrest of dabigatran in a perioperative context. However, the majority of invasive procedures had a low bleeding risk and most patients undergoing high bleeding risk procedures had a dabigatran interruption of only 48 h, which seems insufficient for procedures like neuraxial anesthesia [21, 75–78].

Several observational studies from clinical trials or national registries have shown a higher bleeding risk without reduction of TE with the use of heparin bridging in patients taking DOACs or warfarin [79]. However, in most studies heparin bridging and anticoagulant arrest were not standardized. Furthermore, the invasive procedures had mainly low to moderate bleeding risks and patients at high TE risks were again underrepresented in these trials. Therefore the ongoing PAUSE trial (NCT02228798) which aims to recruit 3300 patients on DOACs in a perioperative setting following a standardized protocol without heparin bridging and with residual DOAC concentration measurements on the day of the invasive procedure, may be able to recruit sufficient high-TE risk patients to strengthen the conclusions of the previous trials. The estimated study completion date is December 2018.

DOACs have a shorter elimination half-life than most VKAs and therefore heparin bridging has no clinical benefit in patients with a short period of perioperative DOAC interruption. The situation might be different for patients with high TE risk and prolonged DOAC arrest (> 72–96 h), e.g. before a neuraxial anesthesia. For prolonged arrest of DOAC, when the risk of TE outweighs the risk of bleeding, patients should benefit from a multidisciplinary management to decide if heparin bridging should be prescribed in order to reduce the perioperative gap without anticoagulant. The use of heparin bridging (LMWH or UFH) requires a clear protocol that should be readily available in each institution. For example, the dose and regimen of LMWH must be adapted according to the patient’s clinical characteristics (e.g. weight and renal function) including the risk of bleeding [80]. Furthermore, both are parenteral anticoagulants which may cause heparin-induced thrombocytopenia and osteoporosis. The use of UFH requires hospitalization to monitor the anticoagulant level, but it has the advantage of being eliminated independently of the patient’s renal function [81]. Indeed, a recent survey demonstrated that despite the evidence that bridging with heparin should be greatly reduced in clinical practice and reserved for particularly high TE settings, many physicians involved in perioperative management still continue to bridge patients with heparin at DOAC arrest without a standardized protocol [19, 33, 45, 46].

### Resuming a DOAC after an invasive procedure or surgery

Advice about DOAC resumption after high bleeding-risk procedures is similar in the different expert guidelines: all recommend that therapeutic doses of DOACs should be deferred for 24–72 h [19, 45, 46]. If necessary, the use of a stepwise approach of bridging therapy with a prophylactic dose of heparin can be considered for patients at high TE risks [33]. Spyropoulos et al. instead propose a reduced dose of dabigatran (75 mg twice daily), rivaroxaban (10 mg once daily) or apixaban (2.5 mg twice daily) on the evening after surgery and on the day after surgery (first postoperative day). They recommend bridging therapy with heparins only in patients who cannot tolerate oral medications [19]. If patients are at high TE risks and hemostasis is not achieved, mechanical VTE prophylaxis should be considered. The GIHP suggest a bridge with heparin by the time an indwelling catheter is in place after neuraxial anesthesia [59].

For prophylactic DOAC resumption in a postoperative context, Rosencher et al. suggest that after neuraxial catheter withdrawal, the anticoagulant can be administered at *“8 hours minus its time to peak concentration (Tmax)”* (which varies from 1 to 4 h in DOACs). They suggest that it takes about 8 h for an initial platelet plug to solidify into a stable clot which will remain intact after administration of anticoagulants [59]. The presence of bleeding during needle puncture or catheter placement should further delay anticoagulant therapy post surgery for 24 h [62].

For pain procedures, the recent ASRA guidelines suggest that the first dose of DOAC can be administrated after an interval of 24 h, unless there is a high risk of VTE. A 12-h interval can be considered in some circumstances, depending on the physician’s judgement [82].

For low bleeding-risk surgery, some experts recommend restarting DOACs 6–8 h after the end of surgery. Spyropoulos et al. recommend waiting 24 h before resuming the full dose of DOAC.

Table 4 describes the main propositions about DOAC resumption in the peri-procedural setting.

Planning safe resumption of DOAC treatment is essential as premature re-initiation of heparin therapy (within 24 h of a procedure) is an avoidable independent predictor of major bleeding [83, 84].

## Doac laboratory testing

DOACs were initially marketed with the advantage of not requiring routine laboratory testing. However, use in frail or obese patients [22], as well as the management of emergencies in patients on DOACs necessitated the development of specific coagulation assays able to answer specific clinical questions accurately.

In the perioperative setting, the 2 main needs are: 1) to exclude clinically relevant concentrations of DOACs before a procedure carrying a high risk of bleeding (e.g. when DOAC interruption has been wrongly assessed or when emergencies require thrombolysis) and 2) to exclude supra-therapeutic plasma concentrations before urgent interventions. In addition, specific plasma levels have been suggested to warrant the administration of DOAC antidotes (i.e. 50 ng/ml for a patient with serious bleeding and 30 ng/ml in patients requiring urgent surgery that cannot be delayed and carries a high risk of bleeding) [85].

Reagents used for routine global assays such as activated partial thromboplastin time (aPTT) for dabigatran and prothrombin time (PT) for direct anti-Xa anticoagulants (rivaroxaban > edoxaban> > apixaban) are not sufficiently accurate to exclude clinically relevant plasma concentrations of DOACs [86–88]. However, both global assays can provide a qualitative assessment of DOACs in the on-therapy range, but their performances depend strongly on the reagent used and for apixaban, even high therapeutic levels may not be detected with PT [89, 90]. In contrast, the thrombin time is very sensitive to the presence of dabigatran and a normal TT excludes this [90]. However, slightly elevated TT does not assess accurately the residual effect of dabigatran due to lack of standardization. Furthermore, the sensitivity of various thrombin reagents can give different TT measurements for the same dabigatran plasma concentration [91–95].

Routine tests are not specific to DOAC and can be prolonged in many situations (e.g. trauma-induced coagulopathy) outside the intake of DOACs. This may lead to incorrect estimation of DOAC anticoagulant level.

For accurate estimation of DOAC plasma concentrations, laboratories must use specific assays with the appropriate methods for the expected DOAC plasma level. The choice of method will depend on the question the clinician needs to answer.

Some specific coagulation assays have adapted calibrators and methods for low plasma DOAC concentrations and these should therefore be used to assess levels <50 ng/ml [95]. These tests can provide accurate estimation in the perioperative setting when clinically relevant DOAC concentrations need to be excluded or when the estimation of DOAC plasma concentrations will guide antidote administration [23].

Importantly, laboratory scientists and clinicians should collaborate to establish an institutional protocol on when and how to test patients on DOACs to highlight what information is needed, to propose the appropriate tests and to provide the correct interpretation of results.

In addition, laboratories need to be informed about any clinical aspects of the patient that might influence the results (e.g. heparin bridging) so they can use the most appropriate test available or to adapt estimates of DOAC plasma concentrations and their significance.

Recently, experts highlighted the urgent need to make accurate, specific coagulation assays widely available [96] and the need for further research to improve the turn-around time of such tests (ideally less than 20 min) to speed up emergency management of patients on DOACs.

### Other options to rapidly screen for the presence of DOACs

When a patient is unconscious and the clinical history is not available, the presence of dabigatran can be excluded if the TT is normal or its presence suspected if the TT is elevated. However, there is no routine assay with similar sensitivity for anti-Xa DOACs (rivaroxaban, apixaban and edoxaban), and therefore some authors reported the use of UFH or LMWH-calibrated heparin assays to screen the presence (or absence) of significant levels of a FXa DOAC. Indeed, LMWH- or UFH-calibrated methods can exclude significant levels of anti-Xa DOACs (< 30 ng/ml) and most coagulation analyzers can run the heparin assays. Furthermore, FDA-approved heparin calibrators and controls are commercially available, which is not the case for anti-Xa DOACs [97–100].

Although heparin assays are not specific and cannot distinguish between heparins and anti-Xa DOACs, their use could assist clinicians in emergency situations where the clinical history is not available (i.e. excluding anti-Xa activity in an unconscious patient who may benefit from thrombolysis).

One promising global assay that could be implemented easily on all coagulometers is the dilute Russell’s Viper Venom Time (dRVV-T). Recent data suggest that this test could provide a rapid estimation of the intensity of anticoagulation with all DOACs without any specific calibrators, and the test can identify sub-therapeutic plasma levels. However, further studies are needed to confirm its clinical utility, as it is currently less widely available than the TT or the anti-Xa chromogenic assays [101].

### Point of care monitoring and other global assays

Point-of-care (POC) monitoring and other global assays (e.g. thrombin generation assay (TGA), prothrombinase induced clotting time (PiCT), thromboelastography (TEG), thromboelastometry (TEM), and activated clotting time (ACT)) have been tested for various DOACs. They can be useful to assess the efficacy of reversal agents. However, they are costly, lack standardization, are insufficiently studied and are not available in routine clinical practice [22, 23, 102]. In addition, they are often not sensitive enough to exclude clinically relevant concentrations of DOACs in the perioperative setting. Their use should therefore be restricted to specific clinical contexts.

## Management of emergencies

Most episodes of bleeding in patients treated with DOACs can be managed with supportive measures and natural clearance of the drug.

A rapid assessment of the patient’s anticoagulation level helps to determine the contribution of the anticoagulant to the bleeding, the need for a reversal strategy and to plan the best timing if an invasive procedure is required [102].

If emergencies require an invasive procedure that can be postponed, the timing for the procedure should be determined from the bleeding risk of the procedure and the residual anticoagulant level of DOACs. The GIHP suggest that a residual DOAC plasma concentration < 30 ng/ml should be reached before undertaking high bleeding risk surgery [102, 103]. A level > 400 ng/ml suggests a high risk of uncontrollable hemorrhage [22].

Recent ISTH guidance for the administration of DOAC antidotes [104] is partly based on DOAC plasma concentration. Experts consider that a threshold ≥50 ng/ml warrants the administration of an antidote in cases of serious bleeding. For urgent invasive procedures carrying a high risk of bleeding, the threshold is ≥30 ng/ml. If specific coagulation assays with calibrators accurate for low DOAC plasma concentrations are not available to guide antidote administration, a normal TT and the absence of anti-Xa activity measured with LMWH- or UFH-calibrated methods can rapidly avoid unnecessary antidote administration.

Patients who have bled may have an acquired coagulopathy (e.g. polytrauma-induced coagulopathy or dilutional coagulopathy) in addition to anticoagulant therapy. Early administration of hemostatic therapy such as prothrombin complex concentrates (PCCs), fibrinogen concentrates, fresh frozen plasma, platelets and antifibrolytics may be critical for preventing complex coagulopathies and progression to severe, life-threatening hemorrhage [105].

In the absence of an antidote, previously suggested reversal strategies can be considered:

- **reduction of intestinal absorption**: activated charcoal should be considered in the first hours after ingestion [106].
- **increase in DOAC clearance:** ensure adequate diuresis, especially for patients taking dabigatran. Only patients on supratherapeutic level of dabigatran may be candidates for renal replacement therapy (RRT) when an invasive procedure needs to be rapidly planned. However, within 4 h after RRT, a rebound of dabigatran plasma concentration can occur with a potential risk of bleeding [104].
- administration of **coagulation factors**: Prothrombin complex concentrates (PCCs) contain lyophilized human plasma-derived vitamin-K dependent coagulation factors (clotting factors II, VII, IX and X) and are standardised according to their factor IX content. They may also contain anticoagulation proteins such as protein C, protein S, protein Z, antithrombin and heparin. They are categorized as 4-factor PCC if their content of FVII is high and as 3-factor PCC if it is low. Furthermore, aPCCs (FEIBA®) are available which contain non-activated factors II, IX and X, and activated factor VII [104]. Prophylactic administration of PCCs is not recommended. The mechanisms of action of PCCs and aPCCs are similar as both increase thrombin generation and animal studies have not shown any significant differences in the reduction of bleeding. Therefore, it is not useful to switch from one to the other when trying to manage bleeding in a patient on dabigatran [45, 103]. In the pre-clinical setting, PCC or aPCC showed an improvement in coagulation parameters, blood loss and mortality. Dose recommendations are difficult to make with the lack of high-level evidence with PCCs and aPCCs for dabigatran reversal, but it appears necessary to use the minimum effective dose because of the theoretical thromboembolic risk (starting with an initial dose of 25 U/kg). Suggested doses for 4-Factor PCCs and aPCC are 50 U/kg and 80 U/kg respectively.

In a recent prospective cohort study of dabigatran-associated major bleeding, the effectiveness of activated prothrombin complex concentrate (median first dose: 44 units/kg, range: 24–98), was assessed as good in 9/14 patients and moderate in 5/14 patients. No thromboembolic events occurred within 30 days [107].

Concerning recombinant factor VIIa (rVIIa), as it did not provide any advantages over PCC or aPCC, recent guidance has withdrawn rVIIa from potential reversal treatment for DOAC in bleeding patients [22].

### Antidotes

The ISTH guidance state that potential indications for antidotes include life-threatening bleeding, bleeding into a critical organ or closed space, prolonged bleeding despite local hemostatic measures, high risk of recurrent bleeding because of overdose or delayed clearance of DOACs, and need for an urgent intervention associated with a high risk of bleeding. Antidotes should not be used when bleeding can be stopped with local hemostatic measures or when interventions can be delayed to enable anticoagulant clearance, especially in patients with normal renal function [108]. Some urgent procedures without bleeding may have antidote administration delayed into the operating room, e.g. for patients with clinically relevant DOAC plasma concentration and unsatisfactory hemostatic conditions.

#### Antidote for dabigatran etexilate

Idarucizumab is a specific reversal agent for dabigatran [108]. It is a humanized mouse monoclonal antibody fragment (Fab) that specifically binds to dabigatran with high affinity (approximately 350-fold greater than the affinity of dabigatran for thrombin) and neutralizes its anticoagulant effect. Idarucizumab, when attached to dabigatran, prevents the latter binding to thrombin. It has an estimated half-life of 45 min [109].

According to the clinical study REVERSE AD Phase III, idarucizumab should be given at a dose of 5 g once via a 5-min intravenous infusion. At this dosage of idarucizumab, the reversibility period of the anticoagulant effect of dabigatran is complete, immediate and sustained [109, 110].

No specific side effects have been attributed to idarucizumab yet, and no changes in coagulation markers in blood tests in the absence of dabigatran [111].

After publication of the first results of the REVERSE AD phase III trial, idarucizumab received an accelerated marketing approval from the FDA and EMA and is now licensed in the United States and European countries for the reversal of the anticoagulant effect of dabigatran in life-threatening or uncontrolled bleeding and urgent surgery or invasive procedures [112, 113].

Some limitations of the REVERSE AD trial should be considered. First, the primary end point was not patients’ clinical outcomes, but normalization of laboratory tests to demonstrate the maximum percentage reversal of the anticoagulant effect of dabigatran (measured with the dilute thrombin time or ecarin clotting time). Two groups of patients on dabigatran were included, 298 patients in group A who had serious bleeding, and 196 patients in group B who required an urgent procedure. The dTT normalized within 4 h in 235/238 patients (98.7%) in group A and 141/143 patients (98.6%) in group B. Clinical outcomes, considered only as secondary endpoints, were assessed by the treating clinician. It is important to note that the median time to the cessation of bleeding in group A was 3.5 h for GI bleeds and 4.5 h for non GI and non ICH bleeds after idarucizumab administration. The authors admitted that this endpoint was difficult to assess in many patients, such as those with intracranial or retroperitoneal bleeding.

Secondly, 51 of the 494 patients who received idarucizumab did not have prolonged dTT in the emergency department, due to dabigatran clearance. Thrombotic events occurred in 31 of 496 patients at 90 days (6.3%). Two thirds received no antithrombotic therapy prior to the event (i.e. VTE, myocardial infarct, ischemic stroke, systemic embolism).

The worrying death rate (18.7% in group A and 18.5% in group B) was, according to the authors, related to the index event or associated with coexisting conditions.

Third, in some patients, there were subsequent increases in dabigatran concentrations 12 h and 24 h after idarucizumab administration. This was probably due to redistribution of extravascular dabigatran into the intravascular compartment, In addition, 7 patients received more than 1 dose of 5 g idarucizumab.

Finally, a major limitation is the lack of a control group with current guidance recommending prothrombin complex concentrates for the management of serious bleeding in dabigatran-treated patients [114].

As this study was not designed to demonstrate a clear benefit in patient outcomes, compared with other reversal strategies, clinicians need to be aware of these limitations and should provide an institutional protocol to guide the administration of idarucizumab and to enable appropriate patient follow-up.

#### Antidote for oral factor Xa inhibitors

Andexanet alpha is a recombinant modified human factor Xa decoy, which is catalytically inactive but able to bind direct factor Xa inhibitors with high affinity at its active site, as well as LMWH or fondaparinux.

Andexanet alpha causes a rapid and reproducible reversal of anticoagulant effects in healthy volunteers receiving rivaroxaban, apixaban, edoxaban or enoxaparin [115]. It has a half-life of approximately 1 h and the maximum effect is achieved after 2–5 min. The dosing strategies used in the phase II clinical trials were different for rivaroxaban (clinical trial ANNEXA-R) and apixaban (clinical trial ANNEXA-A) due to different pharmacokinetic and pharmacodynamic models. An andexanet alpha bolus injection needs to be followed by a continuous intravenous infusion for 2 h due to a rebound effect of factor Xa inhibitors within 15 min of the bolus.

To reverse the effects of rivaroxaban at a daily dose of 20 mg, the recommended bolus of andexanet alpha is 800 mg IV (30 mg per minute) followed by a continuous infusion of 8 mg/min for 2 h (960 mg in total). For apixaban at a dose of 5 mg twice a day, the recommended bolus of andexanet alpha is 400 mg IV (30 mg per minute) followed by a continuous infusion of 4 mg/min for 2 h (480 mg in total).

No serious adverse events or thrombotic complications were recorded in the ANNEXA-A and ANNEXA-R studies, which included 101 patients receiving andexanet alpha.

Recently, results from the phase III trial, the « Prospective, Open-Label Study of Andexanet Alfa in Patients Receiving a Factor Xa Inhibitor Who Have Acute Major Bleeding » (ClinicalTrials.gov number, NCT02329327) have been published. The authors evaluated 67 patients with acute major bleeding within 18 h of the last FXa inhibitor administration. Most bleeds were from gastrointestinal or intracranial sites. Andexanet alpha was administrated in a mean (+ − SD) time of 4.8 h + − 1.8 h after emergency department admission. After bolus administration and the two hour infusion, the median anti-FXa activity decreased by 86% from baseline among patients on rivaroxaban and by 92% among those receiving apixaban. At 12 h after andexanet alpha administration, the median anti-FXa activity had decreased from baseline by 64% for rivaroxaban, and 31% for apixaban. At this time, the clinical hemostasis was estimated as excellent or good in 37 of 47 patients. Rates of excellent or good efficacy occurred in 84% of cases for gastrointestinal bleeding and 80% for intracranial bleeding.

As for idarucizumab, this study also had limitations due to a lack of control group. Furthermore, the death rate was 15% and the high increase of anti-FXa activity at 4 h raises questions of the real contribution of andexanet alpha to the evaluation of the clinical hemostasis. Finally, 18% of patients had an ischemic event during the 30-day follow-up period after the infusion of andexanet. Only 27% of patients resumed anticoagulant therapy within 30 days after acute major bleeding. Birocchi et al. have asked recently if resuming anticoagulant therapy soon after an effective hemostasis could reduce thrombotic events. In conclusion, additional information needs to be collected about the safety of andexanet alpha.

#### Universal antidote

The third approach is the small molecule ciraparantag, which antagonizes the effects of all anticoagulants tested, except VKAs and argatroban. Ciraparantag consists of a small, water-soluble, cationic and synthetic molecule that binds LMWH, UFH, FIIa and FXa inhibitors by non-covalent binding. It can be perceived as a universal antidote for many potential anticoagulant molecules [116]. In vitro studies have shown no major interactions with other coagulation factors or albumin. Animal studies showed a reduction in the blood loss induced by DOACs [117]. In an in vivo study of healthy volunteers who were either untreated or pre-treated with 60 mg of edoxaban, ciraparantag did not induce serious adverse events or a procoagulant signal (measured by D-dimer, TFPI, prothrombin fragments 1.2). A single bolus of 100 to 300 mg of IV ciraparantag normalized the whole blood clotting time to less than 10% above baseline within 30 min in volunteers treated with 60 mg of edoxaban with a stable effect lasting for 24 h. Monitoring the reversal effect of ciraparantag will be challenging in clinical practice as blood collected with sodium citrate, oxalate, EDTA or heparin disrupts the ciraparantag anticoagulant complex and frees the anticoagulant in the plasma. Furthermore, kaolin and celite-based assays are insensitive to monitor the reversal effect of ciraparantag as these activators adsorb the antidote and reduce its active concentration in a blood sample [117].

### Why should we monitor the anticoagulant effect with antidote administration?

The time since last DOAC intake and laboratory tests (including CrCl) can guide clinicians in administering an antidote. The residual anticoagulant effect can only be accurately estimated with specific laboratory tests. However, even if the antidote is administered before the availability of DOAC plasma estimation, understanding the initial concentration at a later time may help to assess the efficacy of the dosage. Indeed, it is important to assess if a single administration is sufficient to decrease the anticoagulant activity to a safe level and to check for potential rebound effects in patients with initial supra-therapeutic DOAC levels or decreased DOAC clearance.

A recent case report described a patient with a plasma dabigatran concentration of 3337.3 ng/ml (assessed by HemosIL® DTI, Instrumentation Laboratory, United States) which decreased to 513.5 ng/ml a few minutes after the administration of 5 g idarucizumab. Seven hours later, the dabigatran level rebounded to 1126 ng/ml.

DOACs distribute within the intra- and extra-vascular compartments, and rebound anticoagulant levels after antidote administration have been described, especially in patients with impaired renal function taking dabigatran. This rebound effect also applies to dabigatran after hemodialysis [118]. In patients with impaired renal function, the reversal of idarucizumab should be monitored after 24 h to exclude dabigatran reappearance if bleeding reoccurs later or an invasive procedure needs to be planned.

Noting the findings from the phase III trial of andexanet alpha, the increase of FXA inhibitor activity 4 h after the end of the infusion should be carefully monitored, especially if patients are still in the operating room for a high risk bleeding procedure, or if they start to bleed again. Interestingly, in 10% of the patients with the highest anti-Xa activity after antidote reversal (median anti-Xa activity 327 ng/ml at the end of the infusion) clinicians considered their clinical hemostasis to be excellent or good [119].

Specific laboratory tests are the only means to distinguish recurrent bleeding due to anti Xa factors or acquired coagulopathy.

In a recent review Greinacher et al. described the potential issues with the development of immunogenicity due to antidote use with DOACs and argue the need to monitor this [120].

Physicians should keep in mind that despite the ability of antidotes to reverse the anticoagulant effects of DOACs, their impact on survival still needs to be proved. Postmarketing registries are needed to determine their clinical utility, especially before thrombolytic therapy in patients with acute ischemic stroke or when additional dosing is necessary due to incomplete reversal and ongoing bleeding [22, 121].

## Conclusions

The correct management of DOACs in the perioperative setting, requires a good understanding of DOAC pharmacokinetics, indications, drug-drug interactions and their effects on laboratory assays. This information should enable clinicians to easily recognize possible problems and solve them.

Decisions in elective situations about when to stop DOACs perioperatively must be based on their half-life, the bleeding risk of the invasive procedures, and on the thromboembolic risk of the patient. Due to the high inter-individual variability of DOAC plasma concentrations, laboratory testing may be useful for specific populations and clinical contexts.

Further perioperative research studies are necessary to confirm previous guidance based on pharmacological and/or laboratory approaches, especially for the management of emergencies or procedures with a high risk of bleeding. The question of whether patients with high TE risks need to be bridged with heparins during prolonged perioperative interruption of DOACs is still not answered. The administration of antidotes needs to be assessed via registries to validate their benefit in outcomes such as survival in patients undergoing emergency procedures with bleeding complications.

## Abbreviations

DOACs: Direct oral anticoagulants
VKAs: Vitamin K antagonists
VTE: Venous thromboembolism
NVAF: Non-valvular atrial fibrillation
CrCl: Creatinine clearance
AF: Atrial fibrillation
TE: Thrombo-embolism
ACCP: American College of Chest Physicians
MHV: Mechanical heart valves
GDF-15: Growth differentiation factor-15
cTnT-hs: high-sensitivity cardiac troponin T
GIHP: Groupe d’Intérêt en Hémostase Péri-opératoire – Working Group on Perioperative Haemostasis
EHRA: European Heart Rhythm Association
MDRD: Modified Diet in Renal Disease
P-gp: P-glycoprotein
ASRA: American Society of Regional Anesthesia
CA: Catheter ablation
LMWH: Low Molecular Weight Heparin
UFH: Unfractionated Heparin
ACT: Activated Clotting Time
ESA: European Society of Anaesthesiology
CI: Confidence interval
OR: Odds ratio
aPTT: Activated partial thromboplastin time
PT: Prothrombin time
TT: Thrombin time
POC: Point-of-care
TGA: Thrombin generation assay
PiCT: Prothrombinase induced clotting time
TEG: Thromboelastography
TEM: Thromboelastometry
dTT: diluted Thrombin Time
DRVVT: Dilute Russell viper venom time
ISTH: International Society of Thrombosis and Haemostasis
ECT: Ecarin clotting time
TFPI: Tissue factor pathway inhibitor
RRT: Renal replacement therapy
PCCs: Prothrombin complex concentrates
aPCC: Activated prothrombin complex concentrates
